# Microwave ablation of hepatocellular carcinoma as first-line treatment: long term outcomes and prognostic factors in 221 patients

**DOI:** 10.1038/srep32728

**Published:** 2016-09-13

**Authors:** Tao Wang, Xiao-Jie Lu, Jia-Chang Chi, Min Ding, Yuan Zhang, Xiao-Yin Tang, Ping Li, Li Zhang, Xiao-Yu Zhang, Bo Zhai

**Affiliations:** 1 Department of Interventional Oncology, Renji Hospital, School of Medicine, Shanghai Jiaotong University, Shanghai, China; 2 Department of Gastroenterology, Shanghai Tongren Hospital, Shanghai Jiao Tong University School of Medicine, Shanghai, China; 3 Department of Statistics, School of Life Sciences, East China Normal University, Shanghai, China; 4 Department of General Surgery, the Affiliated Huai’an Hospital of Xuzhou Medical College and Huai’an Second People’s Hospital, Huai’an, China

## Abstract

This retrospective study aimed at evaluating the long-term outcomes and prognostic factors of microwave ablation (MWA) as a first-line treatment for hepatocellular carcinoma (HCC). 221 consecutive patients receiving MWA in our center between October 11, 2010 and December 31, 2013 were enrolled. Technique effectiveness was evaluated one month post-ablation. Initial complete ablation (CA1^st^) was gained in 201 (90.95%) patients, secondary CA (CA2^nd^) in 8 (3.62%) patients and the remaining 12 (5.43%) patients suffered from incomplete ablation (IA2^nd^) after two sessions of MWA. Patients with tumor size >5 cm were less likely to gain CA1^st^. Procedure-related complications were recorded and no procedure-related death occurred. 22 (10.4%) complications occurred with 8 (3.8%) being major ones. Tumor characteristics (size, number, location) do not significantly influence complication rates. After a median follow-up of 41.0 (ranging 25.0–63.5) months, the median RFS and OS was 14.0 months (95% CI: 9.254–18.746) and 41.0 months (95% CI: 33.741–48.259) respectively. Multivariate analysis identified two significant prognosticators (levels of alpha fetal protein [AFP] and gamma-glutamyl transpeptidase [GGT]) of RFS and five significant prognosticators (tumor number, tumor size, AFP, GGT and recurrence type) of OS. In conclusion, MWA provides high technique effectiveness rate and is well tolerated in patients with HCC as a first-line treatment.

Hepatocellular carcinoma (HCC) is one of the most common cancers and the second leading cause of cancer death worldwide^1^,^2^. Although hepatic resection is still the first line treatment for early-stage HCC patients with well-conserved liver function^3^, thermal ablative therapies have emerged as a well-accepted alternative during recent decades^4^,^5^,^6^,^7^. Thermal ablative therapies destroy tumors either by heating or by freezing within a controllable range^6^,^7^. Among various thermal ablative techniques, radiofrequency ablation (RFA) is currently the most commonly used one and has emerged as a curative treatment for early-stage HCC beyond hepatic resection and liver transplantation^5^,^6^. Microwave ablation (MWA), another thermal ablative technique currently in use, destroy tumors by direct hyperthermia injury similar to RFA^8^. It was reported that the treatment efficacy of MWA is less affected by heat sink effect (vessels near the treated region) compared with that of RFA^6^,^9^,^10^. Recent studies suggested that MWA may be more effective than RFA for large HCC^11^,^12^.

In recent years, MWA is gaining momentum in the clinic. As the number of HCC patients receiving MWA keeps increasing, great variance in the progression-free survival (PFS) and overall survival (OS) after MWA has been observed among individual patients. In order for prognosis predication and patient stratification, there is a need to investigate prognosticators of patients with HCC receiving MWA. This study aimed at evaluating long-term outcomes and complications of HCC patients receiving MWA as an initial treatment and identifying clinicopathologic characteristics that significantly impact patients’ RFS and OS.

## Methods and Materials

### Patient enrollment

**Figure 1:**
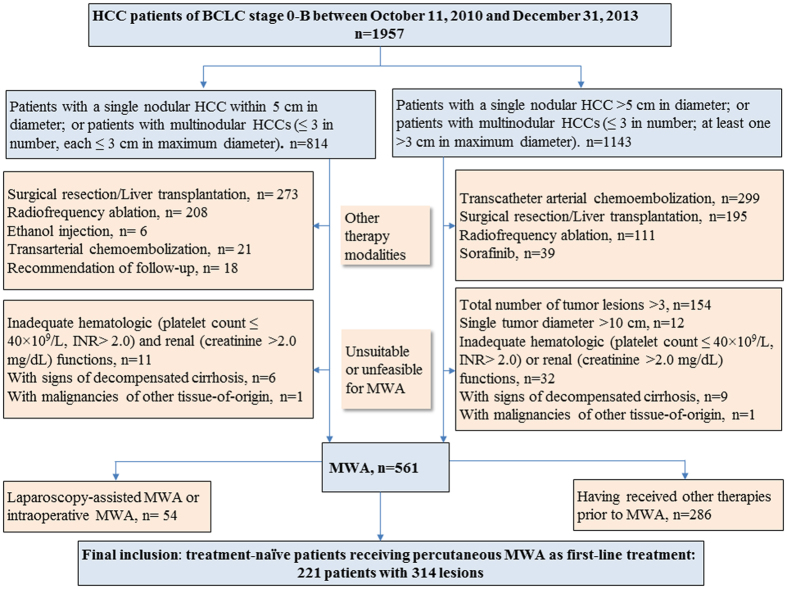
Flowchart of patients enrollment.

The protocol of this study conformed to the ethical guidelines of the World Medical Association Declaration of Helsinki and was approved by the Institutional Ethics Committee of Renji Hospital (Shanghai, China). The medical records of HCC patients who received MWA in Renji Hospital (Shanghai, China) from October 11, 2010 to December 31, 2013 were retrieved and reviewed. Informed consents from patients to allow the review and analyses of their medical records were obtained. The flowchart of patient enrollment of this study is shown in Fig. 1.

Patients inclusion criteria: (1) HCC patients who received ultrasound-guided percutaneous MWA as an initial anticancer treatment; (2) Total number of tumor lesions ≤3; (3) Largest single tumor diameter ≤10 cm; (4) For patients with multiple tumors (2 or 3), no more than one lesion >5 cm; (5) ECOG (Eastern Cooperative Oncology Group) performance status (PST): 0–1; (6) Child-Pugh score A or B; (7) Adequate hematologic (platelet count >40 × 10^9^/L, INR < 2.0) and renal (creatinine <2.0 mg/dL) functions.

Patients exclusion criteria: (1) Patients who received anticancer treatment before MWA, such as hepatic resection, sorafenib, radiofrequency ablation or transcatheter arterial chemoembolization (TACE); (2) Patients who received laparoscopic MWA or intraoperative MWA rather than percutaneous MWA as initial treatment (for tumors protruding from liver surface; for tumors contacting or adhering to diaphragm or abdominal viscera; or for patients with comorbid diseases needing laparoscopy or laparotomy surgery); (3) Patients with portal vein, hepatic vein or inferior vena cava invasion, extrahepatic metastases, or malignancies of other tissue-of-origin; (4) Patients with signs of decompensated cirrhosis such as clinical hepatic encephalopathy and refractory ascites.

### Diagnosis, staging and treatment allocation of HCC

HCC was diagnosed according to the recommendations by the European Association For The Study Of The Liver (EASL)^6^. Briefly, for patients without liver cirrhosis, the diagnosis of HCC was confirmed by biopsies assessed by expert hepatopathologists. For patients with cirrhosis, the diagnosis of HCC require typical features (hypervascular in the arterial phase with washout in the portal venous or delayed phases on multidetector CT scan/dynamic contrast-enhanced MRI) on one imaging technique for nodules >2 cm and on two imaging techniques for those of 1–2 cm. In case of uncertainty or atypical radiological findings, diagnosis was confirmed by biopsy assessed by expert hepatopathologists.

HCC were staged according to the Cancer of the Liver Italian Program (CLIP) stage^13^ and Barcelona Clinic Liver Cancer (BCLC) stage^14^.

For each patient, treatments were allocated based on patient will and clinicopathological characteristics, which were assessed by the HCC Expert Team in Renji Hospital. This team comprised hepatologists, liver surgeons, interventional radiologists and oncologists.

It should be noted that although a substantial part of patients included in our study were candidates for liver resection or liver transplantation according to mainstream guidelines^5^,^6^, they received MWA rather than surgical treatments for the following reasons: (1) refusing surgical treatments for psychological/religious reasons; (2) contraindicated to surgical treatments after assessed by our HCC Expert Team (for example, compromised cardiopulmonary function); (3) unable to receive liver transplantation due to lack of suitable transplant organ or due to economic reasons.

It is also noteworthy that in our institute, the initial treatment options for patients beyond Milan criteria (single tumors ≤5 cm or 3 nodules ≤3 cm) included TACE, MWA, RFA, liver resection, liver transplantation and sorafinib (Fig. 1). There were 55 patients in our study, for example, with tumors >5 cm in diameter. These patients received MWA rather than TACE as an initial treatment because the tumors could be clearly delineated under ultrasound and had a high likelihood to be completely ablated by MWA as assessed by our HCC expert team.

### Procedures and technical parameters of percutaneous MWA under ultrasound guidance

All MWA procedures were performed percutaneously guided by real-time ultrasound (MyLab Twice scanner or HM1498XS1 scanner) using a 3.5 Mhz probe. The selection of local or general anesthesia was based on tumor number, size and location. General anesthesia was performed in patients with tumors ≥3 cm in diameter, multiple tumors, or tumors adjacent to nerve-rich areas such as abdominal wall or major hepatic vessels. Vital signs were monitored throughout the procedures.

MWA was performed with a 2450 MHZ MTC-3C microwave generator (Vision Medical, Nanjing, China), which has a 25 cm cooled-shaft electrode probe (15-gauge) with a 1.5 cm expandable tip. Power output was set at 80 to 100 watts.

During ablation procedure, complete coverage of the tumor region by hyperechoic under real-time ultrasound was regarded as a measure of complete ablation. In non-risk areas (definition shown below), at least 0.5 cm of the normal hepatic parenchyma surrounding the tumor was ablated as an ablative margin to guarantee complete tumor destruction. For tumors in risk areas (definition shown below), the width of ablative margin was narrowed in the intervals between tumor and adjacent tissues to reach a balance between the need of an ablative margin and the avoidance of heat injury to adjacent tissues.

Ablation strategy (Single/multiple electrode, the total number of ablations needed) were discussed at an interdisciplinary meeting prior to each MWA procedure and were dependent mainly on tumor characteristics and patient general conditions.

1. To treat the majority of tumors within 3 cm in diameter, single ablation plus needle-tract ablation with one electrode was usually sufficient, but for those highly irregular ones, multiple ablations were applied so as to guarantee treatment efficacy.
2. For tumors ranging 3–5 cm in diameter, the strategy of multiple overlapping and needle-tract ablations with one electrode were harnessed. The electrode was inserted until the distal margin of the lesion and then was withdrawn every 1.0–1.5 cm to repeat the ablation.
3. For tumors larger than 5 cm, multi-electrode, multi-tract and multiplanar ablation strategy was used in which two electrodes were inserted parallelly (≦3 cm interval) through the same intercostal space to ablate tumor and then repeated the processes through the next intercostal space until the entire tumor was ablated. Detailed ablation parameters were shown in Supplementary Table 1.

After ablation, patients were monitored for a couple of hours in a recovery unit and then were sent back to the ward. A complete panel of blood chemistry including liver and renal functions was examined pre- and post-ablation.

### Definition of risk areas and special ablation techniques for tumors located in these areas

Based on previous literatures^15^,^16^, risk areas were defined as tumor locations within 5 mm of diaphragmatic dome, big vessels (first or second branch of the portal vein, the base of hepatic veins, or the inferior vena cava) or cavity viscera. These areas are regarded as risk areas because percutaneous ablation of tumors in these areas may cause heat injuries to adjacent organs and may be less effective due to heat-sink effect of big vessels^15^,^17^.

It should be noted that in our study, patients with tumors of the following characteristics had already been excluded because what they received were laparoscopy-assisted or intraoperative MWA: tumors protruding from liver surface, contacting or adhering to diaphragm or abdominal viscera.

For tumors in risk areas, specific ablation technique and parameters were used. Optional routes of electrode insertion were carefully considered on ultrasound scrutiny and a route with the least possibility of injuring adjacent tissues or vessels was selected. For tumors adjacent to large vessels, the ablation power was set at relatively low level (for example, 80w) to prevent vessel injury whereas the ablation time (Supplementary Table 1) was elongated to improve technique effectiveness for compensation. For tumors near diaphragmatic dome or cavity viscera (such as gastrointestinal tract and gallbladder), multi-angle ablation strategy with relative low power (80w, for example) and shortened ablation time were harnessed to prevent possible heat injuries to these tissues. For patients (n = 2) with tumors too close to the diaphragm, artificial pleural effusion with 5% glucose was used to separate the lung. Similarly, for patients (n = 3) with tumors too close to the gastrointestinal tract, artificial ascites (5% glucose, 250–3000 mL) was used to separate gastrointestinal tract to prevent heat injury (Supplementary Table 1).

### Technique effectiveness and procedure-related complications

Contrast-enhanced computed tomography (CT) or magnetic resonance (MR) was performed one month after MWA. The ablation was considered complete if the ablation zone completely covered the tumor and if there was no irregular enhancement at the treatment margin, which was recorded as CA1^st^ (complete ablation at first MWA) and regarded as primary technique effectiveness. Otherwise, an additional session of MWA was performed and the patients were reevaluated one month later. If CA was achieved by this time, it was recorded as CA2^nd^ and regarded as secondary technique effectiveness. Otherwise, the treatment was defined as incomplete ablation (IA2^nd^) and technically failing.

According to the recommendations by the International Working Group on Image-Guided Tumor Ablation^18^, major procedure-related complications referred to those that lead to substantial morbidity and disability, increase the level of care, or result in hospital admission or substantially lengthens the hospital stay. All other complications were defined as minor. For patients of CA2^nd^ or IA2^nd^, the complications following the initial and the second sessions of MWA were all recorded for subsequent analysis.

### Patient follow-up and definitions of terminology

Contrast-enhanced CT or MRI and blood chemistry was performed one month after MWA, every 3 months for the first year, and every 4–6 months thereafter.

Tumor progression and recurrence were defined according to recommendations by the International Working Group on Image-Guided Tumor Ablation^18^. In patients with primary or secondary technique effectiveness (CA1^st^ or CA2^nd^), local tumor progression (LTP) was defined as the reappearance of enhancement within or along the margin of the ablation zone on follow-up imagines. Intrahepatic distant recurrence (IDR) was defined as the occurrence of HCC within the liver at locations not contacting the ablation zone. Extrahepatic recurrence (ER) was defined as metastases outside the liver.

Recurrence-free survival (RFS) was calculated from the day of initial MWA to the day of either the earliest event (LTP, IDR, ER or death) or last follow-up without an event. Overall survival (OS) was calculated from the day of initial MWA to the day of death (confirmed by medical records or by family members) or last follow-up.

### Treatment strategy after tumor progression/recurrence

Treatment strategies after tumor recurrence for patients of CA1^st^ or CA2^nd^ included: liver transplantation in 4 patients, hepatic resection in 4 patients, TACE in 86 patients, TACE plus sorafinib in 4 patients, MWA in 20 patients, RFA in 32 patients, and Chinese traditional medicine in 7 patients. Detailed treatment strategies after tumor progression/recurrence were shown in Supplementary Figure 1.

### Statistical Analysis

Chi-square test or Fisher’s exact test were used to analyze the correlations between tumor characteristics and technique effectiveness, as well as correlations between tumor characteristics and incidence of complications. among patients with difference were analyzed with. Median OS (mOS) and median RFS (RFS) were calculated using the Kaplan-Meier method and compared by the log-rank test. Variables with *p* values < 0.15 on univariate analyses were included in multivariate analysis (Cox proportional hazards model; Backward selection). All statistical analyses were conducted using SPSS 22.0 (SPSS 22.0 for Windows, SPSS, Chicago, Illinois, USA). *p* < 0.05 was considered as statistically significant.

## Results

### Patients’ baseline characteristics

According to the patient inclusion and exclusion criteria, a total of 221 patients were included in this study finally (Fig. 1), of which 115 died by the last follow-up (Jan 31, 2016). The median follow-up was 41.0 months (ranging from 25.0 to 63.5 months). A total of 21 items of pre-ablation clinicopathologic features were recorded for subsequent analyses (Table 1).

### Technique effectiveness and long term outcomes

Of these 221 patients, CA1^st^ was gained in 201 (90.95%), CA2^nd^ in 8 (3.62%) whereas the other 12 (5.43%) suffered from technique failure (remaining incomplete ablation [IA2^nd^] after two sessions of MWA). No patients died within 30 days of MWA. Chi-square test or Fisher’s exact test were performed to investigate whether tumor characteristics (size, number and location) can impact technique effectiveness. The results showed that patients with tumor > 5 cm are more likely to suffer from IA2^nd^ and less likely to gain CA1^st^ compared with those with tumor ≤5 cm in size (Table 2). However, tumor number and location have no significant impact on technique effectiveness.

Of the 209 patients with primary or secondary technique effectiveness (CA1^st^ or CA2^nd^), 170 had suffered from tumor progress or recurrence by the last follow-up, of which 33 were LTP, 120 were IDR, and the other 17 were ER. The median RFS (mRFS) and median OS (mOS) of the total cohort was 14.0 months (95% CI: 9.254–18.746) and 41.0 months (95% CI: 33.741–48.259), respectively. The 1- and 2-year RFS rates were 35.9% and 15.3% respectively, and the corresponding OS rates were 87.1% and 63.2% respectively.

### Prognostic factors of recurrence-free survival (RFS)

**Figure 2:**
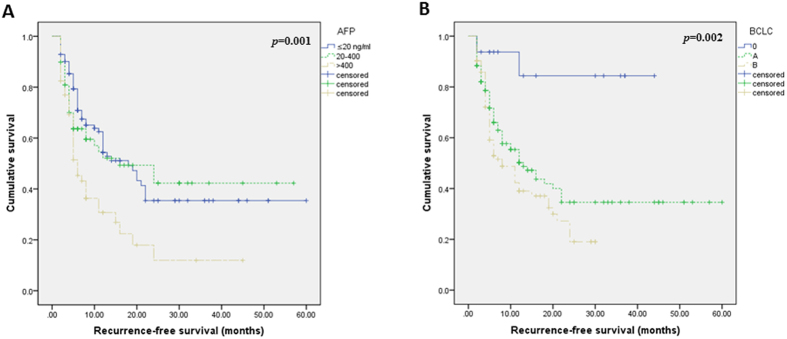
Kaplan-Meier curves of patients’ recurrence-free survival by levels of AFP (**A**) and GGT (**B**). AFP: alpha fetoprotein; GGT: gamma-glutamyl transpeptidase.

Correlations between RFS and 20 dichotomized variables were tested by univariate analyses (Table S2), the results of which showed that the levels of alpha fetoprotein (AFP) and gamma-glutamyl transpeptidase (GGT) significantly correlated with RFS (Fig. 2, Table S2).

On multivariate analyses, levels of AFP and GGT remain significant prognosticators of RFS (Table 3). Compared with AFP > 400 ng/ml, AFP ≤ 20 ng/ml (hazards ratio [HR], 0.532; 95% CI, 0.338–0.837; *p* = 0.006) and AFP 20–400 ng/ml (HR, 0.579; 95% CI, 0.346–0.971; *p* = 0.038) were significant favorable prognosticators of RFS (Table 3). Compared with GGT > 50U/L, GGT ≤ 50 (HR, 0.656; 95% CI, 0.434–0.990; p = 0.045) was significant favorable prognosticators of RFS (Table 3).

### Prognostic factors of overall survival (OS)

**Figure 3:**
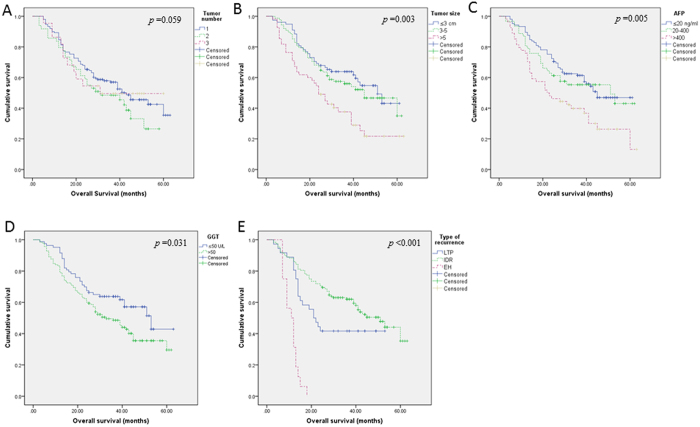
Kaplan-Meier curves of patients’ overall survival by tumor number (**A**), tumor size (**B**), levels of AFP (**C**) and GGT (**D**), and recurrence type (**E**). AFP: alpha fetal protein; GGT: gamma-glutamyl transpeptidase. CA1st: complete ablation at first microwave ablation (MWA); CA2nd: incomplete ablation at first MWA but complete ablation at second MWA; IA2^nd^: remaining incomplete ablation after two sessions of MWA.

On univariate analyses, 5 variables significantly correlated with OS: tumor size, AFP, GGT, technique effectiveness and type of recurrence (Fig. 3, Table S2). For example, the mOS of patients with CA1^st^, CA2^nd^ and IA2^nd^ were 43 months (95% CI: 34.526–62.834), 14 months (95% CI: 0.000–30.631) and 12 months (95% CI: 4.079–19.921) respectively (*p* < 0.001) (Fig. 3).

On multivariate analysis, 6 variables were significant prognosticators of OS: tumor number, tumor size, AFP, GGT and type of recurrence (Table 4, Fig. 3). Compared with one tumor lesion, two lesions were significant unfavorable prognosticator of OS (HR, 3.148; 95% CI, 1.747–5.672; *p* < 0.001), three lesions also showed an unfavorable trend albeit without statistical significance (HR, 2.116; 95% CI, 0.967–4.633; *p* = 0.061). Compared with AFP > 400 ng/ml, AFP ≤ 20 ng/ml (HR, 0.530; 95% CI, 0.323–0.870; *p* = 0.012) and AFP 20–400 ng/ml (HR, 0.565; 95% CI, 0.372–0.977; *p* = 0.041) were significant favorable prognosticators (Table 4). Compared with tumor size >5 cm, tumor size ≤3 cm (HR, 0.106; 95% CI, 0.044–0.255; *p* < 0.001) and 3–5 cm (HR, 0.276; 95% CI, 0.142–0.536; *p* < 0.001) were significant favorable prognosticators. Compared with LTP, IDR (HR, 0.150; 95% CI, 0.174–0.303; *p* < 0.001) was significant favorable prognosticators whereas ER (HR, 6.249; 95% CI, 1.692–23.072; *p* = 0.006) was significant unfavorable prognosticators. Besides, GGT ≤ 50 U/L (HR, 0.332; 95% CI, 0.193–0.572; *p* < 0.001) and ALK ≤ 110 U/L (HR, 0.593; 95% CI, 0.400–0.880; *p* = 0.057) were significant favorable prognositcators of OS relative to GGT > 50 U/L and ALK > 110 U/L, respectively (Table 4, Fig. 3).

### Complications

A total of 22 (10.4%) complications were observed during follow-up, of which 8 (3.8%) were major complications (Table 5). All these complications subsided naturally or after medication. There was no procedure-related death. Chi-square test or Fisher’s exact test showed that tumor size (≤3/3–5/>5 cm), tumor number (1/2/3) and tumor location (risk/non-risk areas) do not significantly influence complication rates (Supplementary Table 3).

## Discussion

To date, only a few studies^15^,^17^,^19^,^20^ have investigated the prognosticators of RFS and OS in patients with HCC who received MWA as an initial treatment, and the majority of these studies suffered from some shortcomings such as small cohort size^15^,^19^,^20^,^21^,^22^, small number of variables to be analyzed^21^,^22^, restriction to tumors ina special range of sizes^15^,^20^,^21^,^22^ or in special areas^17^. Our study, on the contrary, included 221 treatment-naïve patients who received percutaneous MWA as an initial therapy and recorded up to 22 variables for survival analysis. In our study, tumor size ranged from 1 cm to 10 cm, tumor number ranged from 1 to 3, and tumor locations included both risk and non-risk areas. To the best of our knowledge, ours is the first to compressively analyze prognosticators of RFS and OS in HCC patients receiving MWA as an initial treatment.

Our results showed that lower levels of AFP and GGT were significant favorable predictors of RFS on both uni- and multivariate analyses (Table 3, Supplementary Table 2). As for OS, univariate analyses identified 5 significant prognosticators: tumor size, levels of AFP and GGT, technique effectiveness and types of recurrence (Supplementary Table 2). On multivariate analysis, independent significant prognosticators of OS were: tumor number, tumor size, AFP, GGT and types of recurrence (Table 4).

There are several interesting findings in our results that may help to guide patient selection and clinic practice of MWA for HCC. First, although technique effectiveness is not an independent significant prognosticator of OS on multivariate analysis (Table 4), it significantly influence OS on univariate analysis in that the mOS of patients with CR1^st^, CR2^nd^ and IA2^nd^ were 43.0 months (95% CI: 34.526–51.474), 14.0 months (95% CI: 0.000–31.631) and 12.0 months (95% CI: 4.079–19.921), respectively (*p* < 0.001) (Supplementary Table 2). This is in line with the results of previous studies on other percutaneous ablation techniques that highlighted the importance of initial complete response in survival prediction^23^. In some studies^15^,^24^, however, CA1^st^ and CA2^nd^ were both regarded as ablation success whereas IA2^nd^ was regarded as ablation failure. Our study suggests the necessity of future studies to investigate whether it is more rational to regard CA1^st^ as ablation success whereas CA2^nd^ and IA2^nd^ as ablation failure.

Second, our study revealed that MWA can provide complete tumor ablation in the majority of tumors >5 cm (81.8% for CA1^st^ and 3.6% for CA2^nd^). Although tumors >5 cm are not recommended for local ablative therapies in mainstream guidelines^6^, there were 55 patients with tumors >5 cm in our study who received MWA rather than TACE as an initial treatment because these tumors could be clearly delineated under ultrasound and had a high likelihood to be completely ablated by MWA as assessed by our HCC expert team. Our results showed that CA1^st^ was gained in 81.8% of these patients, much higher than the reported initial complete response rate (36.8%, 71/193) of HCC patients receiving repetitive TACE^25^. Therefore, our study overturned a previous assumption that MWA is unable to provide CA for the majority of patients with large HCC. This advance might be attributable to the improvements in MWA equipment and the special techniques we used (multi-electrode, multi-tract and multiplanar ablation strategy) to enable the enlarged ablation range for large HCC. Future perspective studies are needed to compare the long-time efficacy of MWA with those of other therapeutic modalities such as resection or TACE to determine the optimal treatment modalities for this patient population.

Third, tumors located in risk areas (defined exactly as in our study) did not significantly affect RFS, OS and complication rates and should not be regarded as a contraindication to MWA treatment. Controversies long exist regarding the influence of “risk areas” on treatment efficacy of MWA. A previous study^15^, for example, reported that risk areas (using similar definition with ours but not excluding tumors contacting or adhering to neighboring tissues) were independent predicators of tumor recurrence after MWA in a small cohort of HCC patients (n = 45), whereas another study^17^ reported that tumors located within 5 mm of large vessels did not affect patients’ RFS and OS following MWA in a large patient cohort (n = 452). This discrepancy may root in different ablation techniques, different sizes of study cohort, and different definitions of “risk areas”. Interestingly, the results of our study are resonant with a previous study on RFA of HCC in which tumors located in risk areas (using similar definition with ours but not excluding tumors contacting or adhering to neighboring tissues) did not significantly affect LTP and rate of early complications^16^.

Forth, the mOS of patients with LTP (21.0 months; 95% CI: 15.120–26.880) was significant shorter than that of patients with IDR (51.0 months; 95% CI: 39.403–62.597) (*p* < 0.001), lending support to a previous assumption that LTP might be another form of incomplete ablation, in which the residual tumor cells get into a dormant state following ablation and afterwards become the source of recurrence^26^.

However, our study also suffers from some limitations. First, due to its retrospective nature, there were inevitable selection biases in study population. Second, as the vast majority (181/221) of patients in our study had HBV infection, the scenario of antiviral treatments they received may alos impact both RFS and OS and thus should be taken into analyses. However, these data are missing in our study.

In conclusion, our study analyzed long-term outcomes and prognosticators of RFS and OS in HCC patients receiving MWA as an initial treatment. Our results suggest that MWA provides high technique effectiveness rate and is well tolerated in patients with HCC. We identified levels of AFP and GGT as independent prognosticators of RFS and identified tumor number, tumor size, AFP, GGT and recurrence type as independent prognosticators of OS, which may guide patient selection and prognosis prediction and hold the potential of changing clinic practice of MWA for HCC. These results need to be validated in larger patient cohorts, and prognostic nomograms need to be developed in the future to provide reliable and convenient systems for the prediction of RFS and OS in HCC patients receiving MWA.

## Additional Information

**How to cite this article**: Wang, T. *et al*. Microwave ablation of hepatocellular carcinoma as first-line treatment: long term results and prognostic factors in 221 patients. *Sci. Rep.* **6**, 32728; doi: 10.1038/srep32728 (2016).

## Supplementary Material

Supplementary Information

## Figures and Tables

**Table 1:** Patients baseline characteristics.

Clinicopathologic features			Clinicopathologic features		
Age, years	median (range)	58(26–83)	Albumin, g/L	median (range)	40.1 (25.2–51.3)
	<60/≥60	122/99		>35 vs ≤35	182/39
Gender	male/female	178/43	ALT, U/L	median (range)	31 (7–1210)
Etiology	HBV/HCV/others	181/1/39		≤40/>40	143/78
Child score	A/B	193/28	AST, U/L	median (range)	34 (13–743)
Cirrhosis	absent/present	182/39		≤40/>40	139/82
Tumor number	1/2/3	150/49/22	ALK, U/L	median (range)	102 (48–746)
Tumor size, cm	median (range)	4(1–10)		≤110/>110	125/96
	≤3/3–5/>5	78/88/55	GGT, U/L	median (range)	69.25 (8.7–1395.3)
Tumor location	non-risk/risk areas*	129/92		≤50/>50	83/138
AFP, ng/ml	median (range)	23.4(1–50000)	Tbil, μmol/L	median (range)	15.9 (2.4–97.9)
	≤20/20–400/>400	105/62/54		≤25/>25	174/47
PT, sec	median (range)	12(9.1–20.2)	Creatine, μmol/L	median (range)	63 (22.3–144.1)
	≤14/>14	199/22		≤110 vs >110	217/4
INR	median (range)	1.02(0.82–1.67)	CLIP	0/1/2/3	104/86/25/6
	<1.3/≥1.3	214/7	BCLC	0/A/B	16/117/88
Platelets count, 10^9^/L	median (range)	107 (42–432)			
	>100/≤100	117/104			

HBV: hepatitis B virus; HCV: hepatitis C virus; INR: international normalized ratio; AFP: alpha fetal protein; ALT: alanine transaminase; AST: aspartate transaminase; GGT: gamma-glutamyl transpeptidase; PT: prothrombin time; ALK: alkaline phosphatase; TBil: total bilirubin; CLIP: Cancer of the Liver Italian Program; BCLC: Barcelona Clinic Liver Cancer.

^*^Tumors in risk areas refer to those located within 5 mm of diaphragmatic dome, big vessels or cavity viscera, excluding those protruding from liver surface, contacting or adhering to diaphragm, abdominal viscera or big vessels.

**Table 2:** Influences of tumor characteristics on ablation effectiveness.

		CA1st	CA2nd	IA	p value
Total		201(91.0%)	8(3.6%)	12(5.4%)	
Tumor size, cm	≤3	74(94.9%)	3(3.8%)	1(1.3%)	0.782#
	3–5	82(93.2%)	3(3.4%)	3(3.4%)	0.037†
	>5	45(81.8%)	2(3.6%)	8(14.5%)	0.006∮
Tumor number	1	136(90.7%)	4(2.7%)	10(6.7%)	0.669#
	2	46(92.0%)	2(4.0%)	2(4.0%)	0.613†
	3	19(90.5%)	2(9.5%)	0(0.0%)	0.159∮
Tumor location	non-risk	116(89.9%)	5(3.9%)	8(6.2%)	0.876
	risk areas*	85(92.4%)	3(3.3%)	4(4.3%)	

CA1st: complete ablation at first microwave ablation (MWA); CA2nd: incomplete ablation at first MWA but complete ablation at second MWA; IA: remaining incomplete ablation after two sessions of MWA.

^*^Please refer to the legend of Table 1 for the definition of risk areas.

^#^Tumor size ≤3 vs 3–5; tumor number 1 vs 2.

^†^Tumor size 3–5 vs >5; tumor number 2 vs 3.

^∮^Tumor size >5 vs ≤3; tumor number 3 vs 1.

**Table 3:** Multivariate analyses of prognostic factors of recurrence-free survival.

Clinicopathologic features		HR	95% CI	*p* value
Tumor number	1	0.980	0.515–1.866	0.951*
	2	1.546	0.814–2.934	0.320#
	3			
AFP, ng/ml	≤20	0.532	0.338–0.837	0.006*
	20–400	0.579	0.346–0.971	0.038#
	>400			
ALT, U/L	≤40	0.895	0.591–1.354	0.599
	>40			
ALK, U/L	≤110	0.798	0.535–1.190	0.268
	>110			
GGT, U/L	≤50	0.656	0.434–0.990	0.045
	>50			

Variables with p values of <0.25 on univariate analyses were included in multivariate analysis (Cox proportional hazards model). AFP: alpha fetal protein; ALT: alanine transaminase; GGT: gamma-glutamyl transpeptidase; ALK: alkaline phosphatase; TBil: total bilirubin;

^*^Tumor number 1 vs 3.

^#^Tumor number 2 vs 3; They represent the same meaning in other polytomous variables.

**Table 4:** Multivariate analysis of overall survival.

Clinicopathologic features		HR	95% CI	p value
Tumor number	1			
	2	3.148	1.747–5.672	<0.001∮
	3	2.116	0.967–4.633	0.061†
Tumor size, cm	≤3	0.106	0.044–0.255	<0.001*
	3–5	0.276	0.142–0.536	<0.001#
	>5			
AFP, ng/ml	≤20	0.530	0.323–0.870	0.012*
	20–400	0.565	0.372–0.977	0.041^#^
	>400			
ALK, U/L	≤110	0.593	0.400–0.880	0.057
	>110			
GGT, U/L	≤50	0.332	0.193–0.572	<0.001
	>50			
Ablation effectiveness	CA 1^st^	0.499	0.222–0.123	0.093*
	CA 2^nd^	0.517	0.145–1.840	0.308#
	IA			
Types of recurrence	LTP			
	IDR	0.150	0.174–0.303	<0.001∮
	ER	6.249	1.692–23.072	0.006†

Variables with p values of <0.25 on univariate analyses were included in multivariate analysis (Cox proportional hazards model). AFP: alpha fetal protein; GGT: gamma-glutamyl transpeptidase; ALK: alkaline phosphatase; TBil: total bilirubin; CLIP: Cancer of the Liver Italian Program; BCLC: Barcelona Clinic Liver Cancer; CA1^st^: complete ablation at first microwave ablation (MWA); CA2^nd^: incomplete ablation at first MWA but complete ablation at second MWA; IA: remaining incomplete ablation after two sessions of MWA. LTR: local tumor recurrence; IDR: interhepatic distant recurrence; ER: extrahepatic recurrence.

^*^Tumor size ≤3 vs >5 cm; AFP ≤ 20 vs >400 ng/ml; CA1^st^ vs IA.

^#^Tumor size 3–5 vs >5 cm; AFP 20–400 vs >400 ng/ml; CA2^nd^ vs IA.

^∮^Tumor number 2 vs 1; IDR vs LTP.

^†^Tumor number 3 vs 1; ER vs LTP.

**Table 5:** Post-procedure complications after initial and/or second MWA.

	Number of complications	Number of major complications
Pleural effusion	5	1
Ascites	2	0
Intraperitoneal hemorrhage	2	0
Hyperbilirubinemia	5	2
Hepatic encephalopathy	1	1
Renal deficiency	6	3
Adrenal crisis	1	1
Total	22(10.4%)	8(3.8%)
