# Supplementary material for: Microwave ablation of hepatocellular carcinoma as first-line treatment: long term outcomes and prognostic factors in 221 patients
Source: Sci Rep. 2016 Sep 13;6:32728. doi: 10.1038/srep32728 (PMC5020644; doi:10.1038/srep32728)
Supplement: Supplementary Information [file srep32728-s1.doc]

**Supplementary Materials to “Microwave ablation of hepatocellular carcinoma as first-line treatment: long term results and prognostic factors in 221 patients ”**

Tao Wang^1*^, Xiao-Yu Zhang^3*^, Jia-Chang Chi^1^, Min Ding^1^, Li Zhang^4^, Xiao-Jie Lu^2^, Bo Zhai^1^

1.Department of Interventional Oncology, Renji Hospital, School of Medicine, Shanghai Jiaotong University, Shanghai, China.

2.Department of Gastroenterology, Shanghai Tongren Hospital, Shanghai Jiao Tong University School of Medicine, Shanghai, China

3.Department of General Surgery, the Affiliated Huai’an Hospital of Xuzhou Medical College and Huai’an Second People’s Hospital, Huai'an, China.

4.Department of Statistics, School of Life Sciences, East China Normal University, Shanghai, China.

**Table S1. Technical parameters of MWA in 211 patients**

| Ablation time  per tumor | Range | 4-34 min |
| --- | --- | --- |
|  | Mean ± standard deviation | 12.2 ± 6.1 min |
| Ablation  strategy | Single ablation | n=118 |
|  | Multiple overlapping ablation with single electrode | n=127 |
|  | Multiple overlapping ablation with multi-electrode and multiplanar ablation strategy | n=69 |
|  | Total number of HCC lesions | n*=*314 |
| Use of artificial ascites | | n=3 |
| Use of artificial pleural effusion | | n=3 |

MWA: microwave ablation; HCC: hepatocellular carcinoma

**Table S2. Univariate analyses of prognosticators of recurrence-free survival and overall survival.**

| Clinicopathologic features | *p* value | |
| --- | --- | --- |
|  | Recurrence-free survival | Overall survival |
| Age, years, (<60 vs >=60) | 0.406 | 0.319 |
| Gender (male vs female) | 0.577 | 0.496 |
| Etiology (HBV vs HCV vs others) | 0.667 | 0.721 |
| Child score (A vs B) | 0.874 | 0.403 |
| Cirrhosis (absent vs present) | 0.746 | 0.756 |
| Tumor number (1 vs 2 vs 3) | 0.063 | 0.059 |
| Tumor size, cm, (≤3 vs 3-5 vs >5) | 0.540 | 0.003 |
| Tumor location (non-risk vs risk areas^*^) | 0.560 | 0.666 |
| AFP, ng/ml, (≤20 vs 20-400 vs >400) | 0.004 | 0.005 |
| PT, sec, (≤14 vs >14) | 0.995 | 0.700 |
| INR, median (<1.3 vs ≥1.3) | 0.301 | 0.384 |
| Platelets count, 109/L, (>100 vs ≤100) | 0.739 | 0.283 |
| Albumin, g/L, (>35 vs ≤35) | 0.946 | 0.949 |
| ALT, U/L, (≤40 vs >40) | 0.122 | 0.247 |
| AST, U/L, (≤40 vs >40) | 0.309 | 0.219 |
| ALK, U/L, (≤110 vs >110) | 0.089 | 0.075 |
| GGT, U/L, (≤50 vs >50) | 0.026 | 0.031 |
| Tbil, μmol/L, (≤25 vs >25) | 0.803 | 0.214 |
| Creatine, μmol/L, (≤110 vs >110) | 0.428 | 0.397 |
| Technique effectiveness (CA1st vs CA2nd) | 0.608 |  |
| Technique effectiveness (CA1st vs CA 2nd vs IA) | NA | <0.001 |
| Types of recurrence (LTP vs IDR vs ER) | NA | <0.001 |

HBV: hepatitis B virus; HCV: hepatitis C virus; INR: international normalized ratio; AFP: alpha fetal protein; ALT: alanine transaminase; AST: aspartate transaminase; GGT: gamma-glutamyl transpeptidase; PT: prothrombin time; ALK: alkaline phosphatase; TBil: total bilirubin; CLIP: Cancer of the Liver Italian Program; BCLC: Barcelona Clinic Liver Cancer; CA1st: complete ablation at first microwave ablation (MWA); CA2nd: incomplete ablation at first MWA but complete ablation at second MWA; IA: remaining incomplete ablation after two sessions of MWA; LTR: local tumor recurrence; IDR: interhepatic distant recurrence; ER: extrahepatic recurrence. *: Tumors in risk areas refer to those located within 5 mm of diaphragmatic dome, big vessels or cavity viscera, excluding those protruding from liver surface, contacting or adhering to diaphragm, or abdominal viscera.

**Table S3. Correlations between tumor characteristics and complication rates**

|  | | Complications | | *p* value |
| --- | --- | --- | --- | --- |
|  |  | Yes | No |  |
| Tumor size, cm | ≤3 | 9(11.5%) | 69 | 0.716 |
|  | 3-5 | 9(10.2%) | 79 |  |
|  | >5 | 4(7.3%) | 51 |  |
| Tumor number | 1 | 12(8.0%) | 138 | 0.224 |
|  | 2 | 8(16.3%) | 41 |  |
|  | 3 | 2(9.1%) | 20 |  |
| Tumor location | non-risk | 16(12.4%) | 113 | 0.15 |
|  | risk areas* | 6(6.5%) | 86 |  |

*: Tumors in risk areas refer to those located within 5 mm of diaphragmatic dome, big vessels or cavity viscera, excluding those protruding from liver surface, contacting or adhering to diaphragm or abdominal viscera.

**
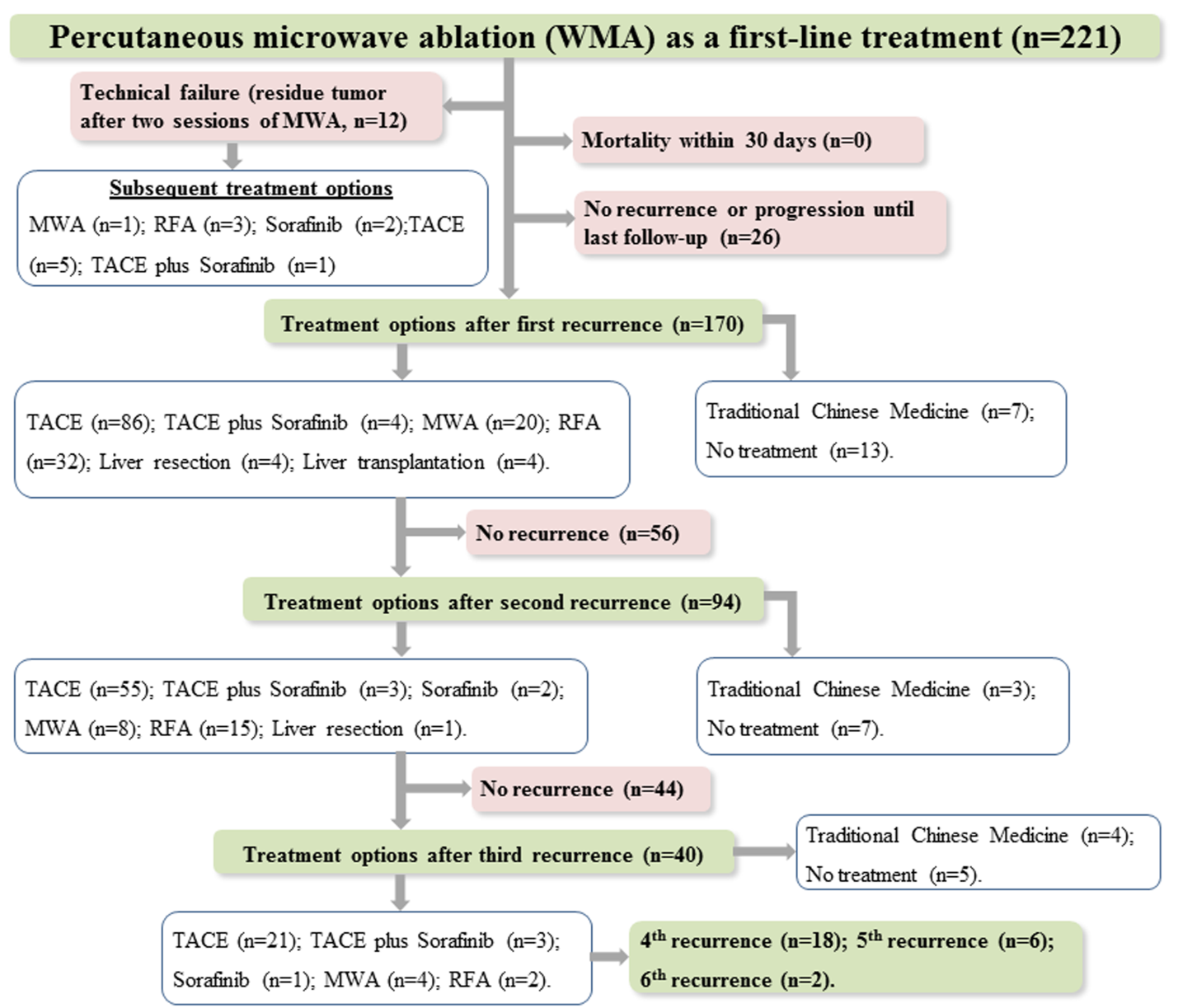
**

**Figure S1. Treatment strategies after recurrence in patients with primary or secondary technique effectiveness.**
